# Mental health challenges, treatment experiences, and care needs of post-secondary students: a cross-sectional mixed-methods study

**DOI:** 10.1186/s12889-023-15452-x

**Published:** 2023-04-06

**Authors:** Elnaz Moghimi, Callum Stephenson, Gilmar Gutierrez, Jasleen Jagayat, Gina Layzell, Charmy Patel, Amber McCart, Cynthia Gibney, Caryn Langstaff, Oyedeji Ayonrinde, Sarosh Khalid-Khan, Roumen Milev, Erna Snelgrove-Clarke, Claudio Soares, Mohsen Omrani, Nazanin Alavi

**Affiliations:** 1 Department of Psychiatry, Faculty of Health Sciences, Queen’s University, 166 Brock Street, Kingston, ON K7L 5G2 Canada; 2 Waypoint Research Institute, Waypoint Centre for Mental Health Care, Penetanguishene, Canada; 3 Centre for Neuroscience Studies, Faculty of Health Sciences, Queen’s University, Kingston, ON Canada; 4 Student Wellness Services, Queen’s University, Kingston, ON Canada; 5 Wellness, Accessibility & Student Success, St. Lawrence College, Kingston, ON Canada; 6 School of Nursing, Faculty of Health Sciences, Queen’s University, Kingston, ON Canada; 7 OPTT Inc, Toronto, ON Canada

**Keywords:** Mental health, Student mental health, Mental health treatment, Psychotherapy, Post-secondary students, Mental health needs, University and college mental health, Young-adult mental health, Treatment perception, Online mental health treatment

## Abstract

**Background:**

Post-secondary students frequently experience high rates of mental health challenges. However, they present meagre rates of treatment-seeking behaviours. This elevated prevalence of mental health problems, particularly after the COVID-19 pandemic, can lead to distress, poor academic performance, and lower job prospects following the completion of education. To address the needs of this population, it is important to understand students' perceptions of mental health and the barriers preventing or limiting their access to care.

**Methods:**

A broad-scoping online survey was publicly distributed to post-secondary students, collecting demographic, sociocultural, economic, and educational information while assessing various components of mental health.

**Results:**

In total, 448 students across post-secondary institutions in Ontario, Canada, responded to the survey. Over a third (*n* = 170; 38.6%) of respondents reported a formal mental health diagnosis. Depression and generalized anxiety disorder were the most commonly reported diagnoses. Most respondents felt that post-secondary students did not have good mental health (*n* = 253; 60.5%) and had inadequate coping strategies (*n* = 261; 62.4%). The most frequently reported barriers to care were financial (*n* = 214; 50.5%), long wait times (*n* = 202; 47.6%), insufficient resources (*n* = 165; 38.9%), time constraints (*n* = 148; 34.9%), stigma (*n* = 133; 31.4%), cultural barriers (*n* = 108; 25.5%), and past negative experiences with mental health care (*n* = 86; 20.3%). The majority of students felt their post-secondary institution needed to increase awareness (*n* = 231; 56.5%) and mental health resources (*n* = 306; 73.2%). Most viewed in-person therapy and online care with a therapist as more helpful than self-guided online care. However, there was uncertainty about the helpfulness and accessibility of different forms of treatment, including online interventions. The qualitative findings highlighted the need for personal strategies, mental health education and awareness, and institutional support and services.

**Conclusions:**

Various barriers to care, perceived lack of resources, and low knowledge of available interventions may contribute to compromised mental health in post-secondary students. The survey findings indicate that upstream approaches such as integrating mental health education for students may address the varying needs of this critical population. Therapist-involved online mental health interventions may be a promising solution to address accessibility issues.

**Supplementary Information:**

The online version contains supplementary material available at 10.1186/s12889-023-15452-x.

## Background

Post-secondary students frequently report high levels of stress, anxiety, depression, and other mental health concerns [1–3]. In a national survey of Canadian post-secondary students using campus mental health services, 95% reported being overwhelmed and exhausted, 83.7% reported anxiety, 86% were depressed, and 81% experienced loneliness [4]. Further, 45.1% of post-secondary students experience higher than average stress levels, and up to 35% meet diagnostic criteria for at least one mental health disorder [5, 6]. Compromised mental health may increase student distress, academic probations, dropouts, and challenges in finding future employment [2, 7–9].

The importance of effective mental health services for all citizens, including youth and young adults is broadly recognized in Canadian society [10]. As a result, most postsecondary institutions offer several different mental health services to vulnerable groups. These may include social or peer support, promotion and outreach programs, health education, counselling services, accommodations, triage system for urgent care, and short-term therapy [11, 12]. Campuses typically do not offer long-term therapy or off-site services [11]. Despite the services available, treatment-seeking is reported to be as low as 10% [5, 13, 14]. Factors such as cost, stigma, privacy concerns, heavy academic course loads, inability to distinguish between mental illness and stress or other common emotions, work priorities, and long wait times can deter students from seeking help [14, 15]. Moreover, adequate and timely access to mental health services by students is adversely affected by a network of personal, social, cultural, and institutional barriers that vary between regions and individuals [15]. These barriers include limited resources, increased complexity in student psychopathology, fragmented services, and low funding, to name a few [11, 16]. Indeed, many student counselling centres report increased service wait times and reduced therapy sessions [11]. These strains are worsened in smaller institutions with fewer staff and resources [17]. Treatment barriers and service use amid the COVID-19 pandemic have yet to be fully delineated.

Mental health concerns were exacerbated by the COVID-19 pandemic, affecting all profiles of post-secondary students [18]. In a recent qualitative study, students without pre-existing mental health conditions reported greater social and academic isolation than those with pre-existing mental health concerns [19]. Furthermore, a recent meta-analysis noted an increase in the prevalence of anxiety (36%) and depression (39%) during the pandemic [20]. In response, many institutions have shifted to telemental health services that encourage continued care [21]. Other universities have started to implement virtual interventions to help first-year, graduate, and professional students navigate through the changed academic and social environments [22, 23]. When assessing support offered by institutions during prolonged campus lockdowns, 91% of Canadian post-secondary institutions offered virtual counselling services, and 84% provided general psychoeducational resources [24]. Similar services are also available in US-based institutions [25]. Nevertheless, poor wellness behaviours, mood, and attention in post-secondary students have been observed during the pandemic [26]. To better understand the efficacy and effectiveness of the available resources during this period, it is critical to investigate how students perceive their mental health challenges and use the services at hand.

The current study aimed to capture post-secondary student mental health and care needs during the post-lockdown period of the COVID-19 pandemic. Specifically, the study surveyed post-secondary students on their mental health knowledge, treatment-seeking behaviours, perception of mental health services, and perceived barriers that may have limited their access to care. Since the persistence of lockdowns and social distancing laws resulted in online therapy use becoming commonplace, this delivery format was also focused on. The findings of this needs assessment study can inform the development of targeted, effective, and equitable care initiatives for this population.

## Methods

### Study design

The following cross-sectional mixed-methods study collected data through a self-administered online survey. A broad-scoping mental health experience survey was publicly advertised to post-secondary students across Ontario, Canada, through social media. Paid advertisement was not utilized for this study and organic reach was obtained by posting to groups and pages that pertained to the target population. Specifically, the survey advertisement was posted on Ontario-based university and college groups and pages on Reddit and Facebook. The research team as well as Queen’s University health and wellness centres also posted the study advertisement on their Instagram, Reddit, and Facebook social media pages. Data collection occurred from May 5 to June 5, 2022. Participation was voluntary, and the survey was administered via Qualtrics (Qualtrics, Provo, UT). Post-secondary students enrolled in any public or private post-secondary institution in Ontario, Canada, were eligible to complete the survey, which entered them in a draw to receive 1 of 20 Amazon gift cards valued at $25. Students of any status, including international, domestic, part-time, full-time, certificate, or degree, were included. To verify student status, participants were asked to provide an institutional email after consenting to participate in the study. The survey link was sent to this email. The consent process informed the students that their responses were in no way linked to their institutional emails. Participants were also asked to include the name of their post-secondary institution in their survey responses. Any student involved in developing or testing the survey was not eligible to complete the survey. Ethics approval was obtained from the Queen’s University Health Sciences and Affiliated Teaching Hospitals Research Ethics Board (HSREB) in Kingston, Canada.

### Survey development

The research team developed and disseminated the *Post-Secondary Student Mental Health Experience Survey* to all post-secondary students attending colleges or universities in Ontario, Canada. The decision to limit the survey to Ontario was to pilot the survey and make any necessary modifications before administering the survey to a broader population [27]. Before dissemination, the survey was reviewed with a sample of post-secondary students enrolled in different programs (*n* = 10) and modifications were made based on their feedback. These students were highly involved in student affairs and leadership positions within their post-secondary institutions. The diverse sample also represented an array of equity-deserving communities, including BIPOC, 2SLGBTQ + , international students, and students with different physical and mental health conditions. The survey was then uploaded onto Qualtrics (Qualtrics, Provo, UT) and was tested by another sample of students (*n* = 20) before finalization. All student reviewers were within the research team’s broad network and agreed to review the survey voluntarily. Once the necessary changes were made, the survey was made available to all post-secondary students attending colleges or universities in Ontario, except for those involved in the survey's development and testing. All participants provided informed consent online before accessing the survey. The final survey focused on specific demographic variables, knowledge of and experience with different mental health treatments and resources (including online delivery formats), perceptions of the accessibility of the current mental health treatments, motivation and likelihood of seeking various types of mental healthcare, and changes students would like to see in the current system of care (Additional file 1). The survey intended to capture post-secondary students' mental health needs and identify barriers and limitations to care.

### Data analysis

Since participants could skip questions they preferred not to answer, data from participants who did not complete the survey in its entirety were also included in the analysis. Missing data were not imputed because the sample size was sufficient to conduct the descriptive analysis. Each item was assessed individually and reported count percentages were relevant to the total responses of each item. All descriptive analyses were conducted through the online Qualtrics (Qualtrics, Provo, UT) statistical analysis software. Data from the open-ended question at the end of the survey (*Do you have any additional feedback to share*?) were analyzed qualitatively using content analysis methods [28, 29]. All relevant feedback was categorized under codes representing prevalent ideas and common categories across the responses. The codes were meant to provide more context on the research findings—in particular, assessing the mental health challenges and care needs of post-secondary students. In the first step, a conventional coding approach was used where coding categories were developed from direct analysis of the entire textual data [30]. To do this, participant feedbacks were read several times to capture a general sense of the data. Subsequently, primary codes explicitly representing the participant responses were developed and modified. Data coding was done semantically, staying close to the text and using the participants’ words and verbiage. After discussion and refinement by the research team, the final coding strategy was applied to the dataset by two independent coders (EM and CaS), and inter-coder reliability was assessed via Cohen’s Kappa. Before finalization, all discrepancies were resolved through a third coder (GG). A subcategory analysis was then conducted by one of the coders (EM), and any emergent secondary codes were finalized through discussion with the research team. The demographic characteristics of participants who provided qualitative data were compared to that of all survey respondents via chi-squared tests for categorical variables and independent sample t-tests for continuous variables at α = 0.05. All statistical analysis was conducted using IBM SPSS Statistics for Mac, version 24 (IBM Corp., Armonk, N.Y., USA).

## Results

### Participants

In total, 448 responses were recorded from students across post-secondary institutions in Ontario, Canada. Participants who did not list their post-secondary institution or if the institution was not located in Ontario, Canada, were excluded (*n* = 78). On average, participants were 21.9 years of age (SD = 4.1). Most respondents did not have children (*n* = 437; 97.5%). Participants reporting their race or ethnicity were given the option to select all applicable identities. For demographic information, please see Table 1.

**Table 1:** Demographic data of survey respondents

Category	*n* (%)
**Gender Identity**	
*Woman*	267 (59.6)
*Man*	160 (35.7)
*Nonbinary*	15 (3.3)
*Prefer to self-describe*	2 (0.4)
*Prefer not to answer*	4 (0.9)
**Biological Sex**	
*Female*	281 (62.7)
*Male*	160 (35.7)
*Intersex*	1 (0.2)
*Prefer not to answer*	6 (1.3)
**Sexual Orientation**	
*Straight/Heterosexual*	321 (71.7)
*Bisexual/Polysexual*	60 (13.4)
*Gay/Lesbian*	18 (4.0)
*Asexual*	10 (2.2)
*Other*	15 (3.3)
*Prefer not to answer*	24 (5.4)
**Preferred Pronouns**	
*She/Her*	253 (56.5)
*He/Him*	155 (34.6)
*They/Them*	12 (2.7)
*Other*	9 (2.0)
*Prefer not to answer*	19 (4.2)
**Race/Ethnicity**	
*White (European descent)*	236 (52.7)
*Black (African, African Canadian, Afro-Caribbean Descent)*	63 (14.1)
*South Asian (Bangladeshi, Indian, Indo-Caribbean, Pakistani,* *Sri Lankan descent)*	56 (12.5)
*East Asian (Chinese, Japanese, Korean, Taiwanese Descent)*	53 (11.8)
*Middle Eastern (Arab, Persian, Afghan, Egyptian, Kurdish,* *Lebanese, Turkish descent)*	20 (4.5)
*Latin American (Hispanic or Latin American descent)*	13 (2.9)
*Southeast Asian (Cambodian, Filipino, Indonesian, Thai,* *Vietnamese descent)*	11 (2.5)
*Indigenous (First Nation, Inuk/Inuit, Métis descent)*	7 (1.6)
*Multi-ethnic*	7 (1.6)
*Other*	6 (1.3)
*Do not know*	1 (0.2)
*Prefer not to answer*	18 (4.0)
**Relationship Status**	
*Single, never been in a romantic relationship*	138 (30.8)
*Exclusive romantic relationship (not married)*	109 (24.3)
*Single, in a serious romantic relationship previously*	90 (20.1)
*Casually dating*	41 (9.2)
*Married*	17 (3.8)
*Common-law*	17 (3.8)
*Nonexclusive romantic relationship (not married)*	6 (1.3)
*Divorced*	2 (0.4)
*Other*	5 (1.1)
*Prefer not to answer*	23 (5.1)

### Post-secondary student profile

**Fig. 1:**
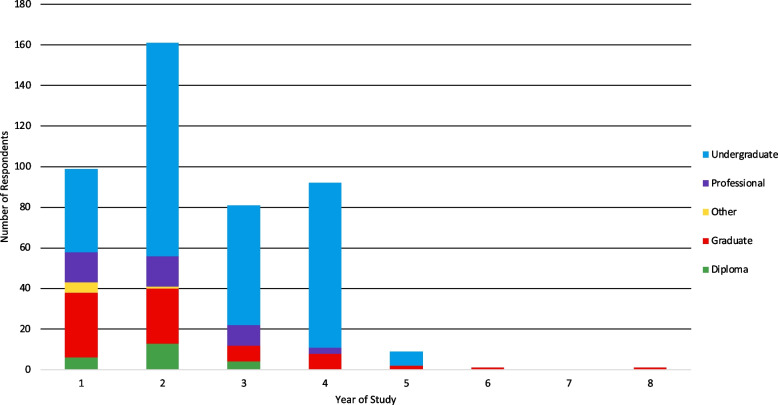
Year of study and degree type of students

The study respondents were enrolled in 34 different institutions across Ontario. Only one participant attended a private institution (Additional file 1). Respondents were mainly enrolled in universities (*n* = 406; 90.6%) compared to colleges (*n* = 42; 9.4%). The majority of university students attended Queen’s University (*n* = 80; 17.9%), followed by the University of Toronto (*n* = 43; 9.6%), Ontario Tech University (*n* = 38; 8.5%), Carleton University (*n* = 37; 8.4%), McMaster University (*n* = 37; 8.3%), University of Waterloo (*n* = 35; 7.8%), and York University (*n* = 21; 4.7%). Most of the respondents attending college were enrolled at St. Lawrence College (*n* = 12; 2.7%), Durham College (*n* = 6; 1.3%), and Centennial College (*n* = 4; 0.9%). Regarding degree type, 65% of respondents (*n* = 290) were undergraduate students, and 17.9% (*n* = 80) were graduate students. Further, 9.8% (*n* = 44) of respondents were pursuing a professional degree, diploma (*n* = 24, 5.4%), or other (*n* = 9; 2%; Fig. 1). Most students were Ontario residents (*n* = 351; 90.5%) and did not relocate from another province. Employment status was nearly equal among students, with 48.8% of respondents being employed (*n* = 217) and 51.2% (*n* = 228) not employed. Students who indicated their weekly employment hours (*n* = 73) worked an average of 25.87 h per week (SD = 11.76). Students (*n* = 77) also committed an average of 5.4 h (SD = 5.24) to weekly volunteering. See Table 2 for a summary of student profiles.

**Table 2:** Participant student profiles

Category	*n* (%)
**Degree Type**	
*Undergraduate*	291 (65.0)
*Graduate*	80 (17.9)
*Professional*	44 (9.8)
*Diploma*	24 (5.4)
*Other*	9 (2.0)
**Enrolment Status**	
*Full-time*	396 (88.4)
*Part-time*	36 (8.0)
*On leave*	8 (1.8)
*Other*	8 (1.8)
**Student Status**	
*Domestic (Canada)*	389 (86.8)
*International*	59 (13.2)
**Housing Arrangement**	
*Living with family/parents*	161 (36.2)
*Living with housemates*	127 (28.5)
*Living on campus residence*	75 (16.9)
*Living with significant other*	38 (8.5)
*Living alone*	35 (7.9)
*Other*	9 (2.0)

### Mental health disorders and symptoms

**Fig. 2:**
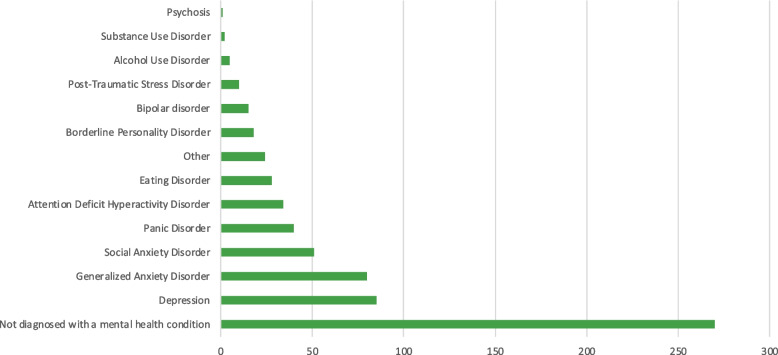
Prevalence of mental health disorders among participants

Most participants reported not being diagnosed with a mental health disorder (*n* = 270; 61.4%; Fig. 2). Amongst those diagnosed, depression (*n* = 85; 19.3%) and generalized anxiety disorder (*n* = 80; 18.2%) were the most prevalent, followed by social anxiety disorder (*n* = 51; 11.6%), panic disorder (*n* = 40; 9.1%), and attention deficit hyperactivity disorder (*n* = 34; 7.7%). Participants who were diagnosed with other mental health disorders (*n* = 24; 5.5%), most frequently reported obsessive–compulsive disorder (*n* = 9; 37.5%). Most participants (*n* = 351; 80.7%) reported not having a disability. The most frequently reported disability was neurodevelopmental (*n* = 36; 8.6%). When asked if students experienced a decline in their mental health since starting their post-secondary education, 66.5% of respondents (*n* = 290) reported a decline in their mental health, 23.4% (*n* = 102) did not see a difference in their mental health, and 10.1% (*n* = 44) were unsure. Specifically, 66.1% of students (*n* = 288) reported problems concentrating, 59.6% (*n* = 260) experienced symptoms of depression (i.e., low moods, low energy, and low motivation), 58% (*n* = 253) experienced daily general anxiety, 51.4% (*n* = 244) experienced anxiety in social situations, 42.2% (*n* = 184) had mood swings, 33.7% (*n* = 147) experienced panic attacks, and 13.5% (*n* = 59) engaged in problematic use of alcohol or other substances including cannabis. Other symptoms expressed by students (*n* = 13; 3.0%) predominately comprised insomnia and other sleep disturbances (*n* = 5; 3.8%).

### Mental health needs

Although most students indicated that their mental health knowledge was good (*n* = 283; 67.7%), the majority (*n* = 261; 62.4%) agreed with the statement that they did not have enough coping strategies and tools related to mental health when starting their post-secondary studies. Many did not believe that post-secondary students have good mental health (*n* = 253; 60.5%). Furthermore, not having enough time to focus on mental health during post-secondary studies was also expressed as a concern by participants (*n* = 198; 47.3%). Most respondents (*n* = 278; 66.5%) believed that the majority of students keep their mental health problems a secret. With respect to high school education, most (*n* = 209; 50%) did not believe that the awareness and education on mental health were adequate, and others were neutral about this sentiment (*n* = 71; 17%). On the other hand, many participants (*n* = 307; 73.5%) believed that improving high school students’ mental health could enhance their overall functioning and well-being during their post-secondary studies.

### Treatment-seeking behaviours

Concerning treatment, 70.5% (*n* = 182) of respondents indicated that they were not taking medication, and 71.5% (*n* = 318) were not receiving counselling or psychotherapy for their mental health. Amongst those receiving counselling or psychotherapy, the majority (52.0%; *n* = 66) received their sessions over online video platforms and 33.9% (*n* = 43) engaged in in-person sessions. Many participants believed that digital mental health care delivery was good but not as good as in-person delivery (*n* = 132; 31.1%), 18.6% (*n* = 79) were unsure, 12.3% (*n* = 52) believed it was no different, and 9.0% (*n* = 38) believed it was better.

**Fig. 3:**
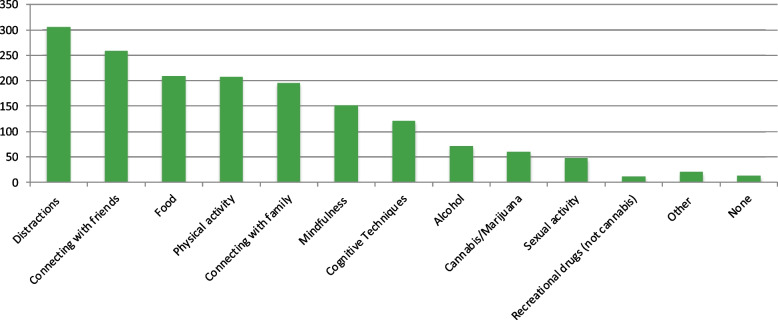
Strategies used to cope with stress

When asked about the campus environment and resources, most participants (*n* = 321; 73.6%) indicated that they had not experienced any form of harassment on campus. However, 17.9% (*n* = 78) of participants experienced verbal harassment, followed by cyber harassment (*n* = 27; 6.2%), sexual harassment (*n* = 25; 5.7%), and physical harassment (*n* = 22; 5.0%). Most students (*n* = 267; 61.7%) did not have accommodations such as extra test time or separate exam rooms. Some students (*n* = 151; 34.9%) reported not using mental health services during their post-secondary studies. However, 34.6% (*n* = 150) of students used the student wellness services offered by their institutions, and 25.4% (*n* = 110) used mental health services provided outside their post-secondary institution. Participants also used several different strategies to cope with stress (Fig. 3). The most frequently used approach was distractive behaviours such as hobbies (*n* = 305; 70.4%), followed by connecting with friends (*n* = 258; 59.6%), food (*n* = 209; 48.3%), and physical activity (*n* = 208; 48.0%).

**Fig. 4:**
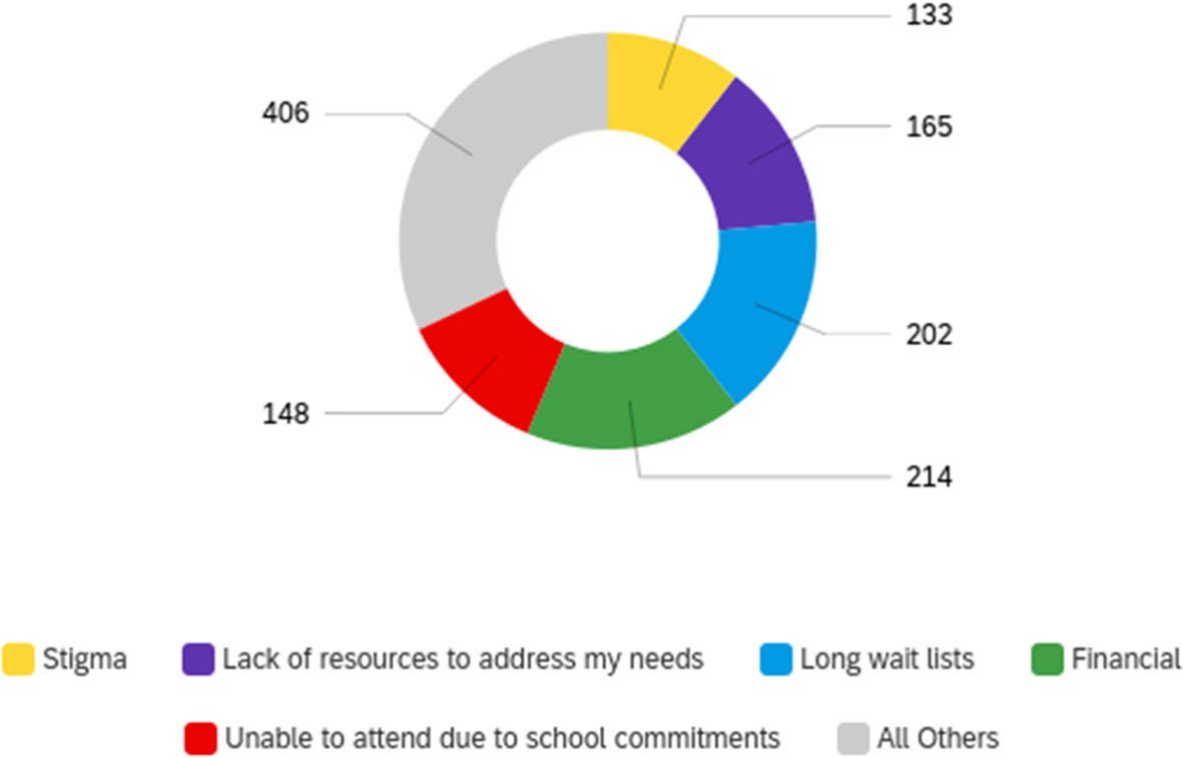
Perceived barriers to care (response count)

The most frequently reported barriers to mental health care were financial (*n* = 214; 50.5%), long wait lists (*n* = 202; 47.6%), lack of resources to address needs (*n* = 165; 38.9%), inability to receive care due to school commitments (*n* = 148; 34.9%), stigma (*n* = 133; 31.4%), cultural barriers (*n* = 108; 25.4%), and past negative experiences with mental health care (*n* = 86; 20.3%). Only 8.3% (*n* = 35) of respondents indicated no barriers to receiving care. Respondents who indicated “other” (5.0%, *n* = 21) listed accessibility issues (*n* = 4; 19.0%), anxiety or social anxiety preventing treatment-seeking (*n* = 2; 9.5%), work commitments (*n* = 2), and apathy (*n* = 2; Fig. 4).

### Mental health care

**Fig. 5:**
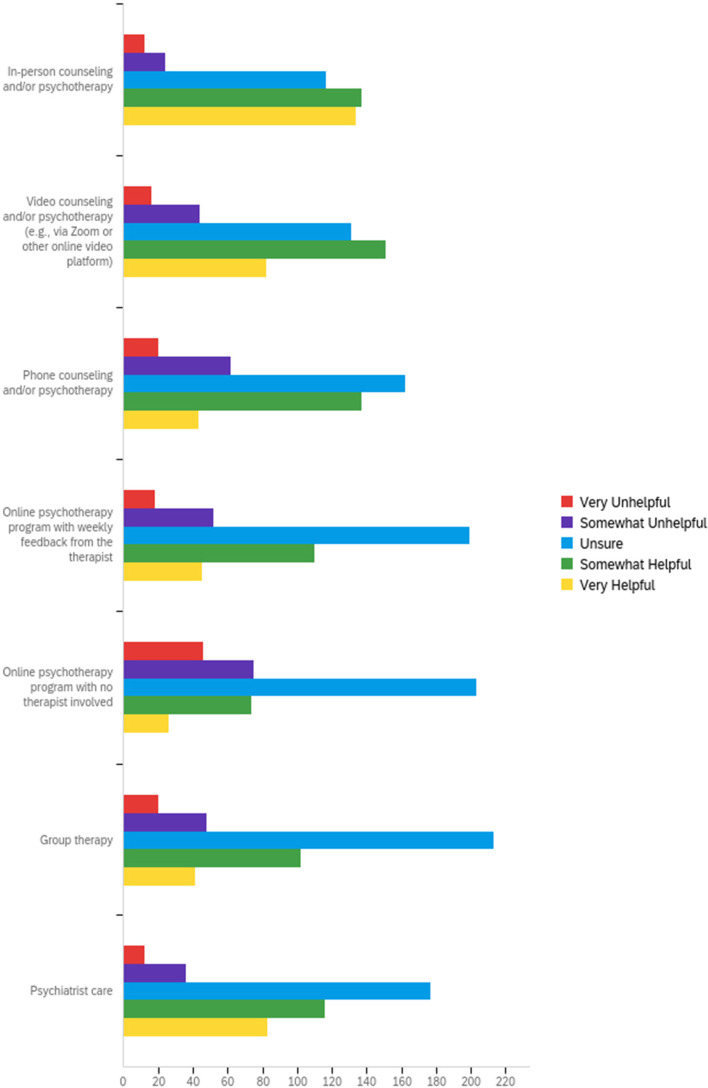
Perception of care helpfulness

**Fig. 6:**
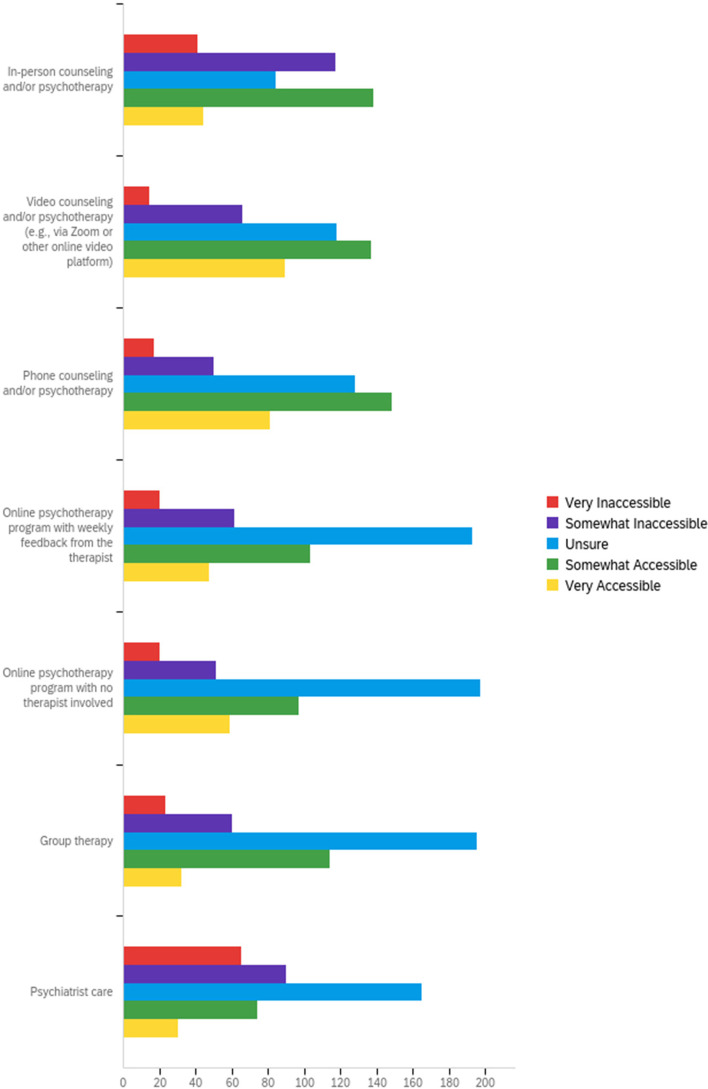
Perception of care accessibility

A sizeable proportion of participants were unsure about the helpfulness of different forms of mental health care (Fig. 5). Although in-person services were frequently rated as helpful (*n* = 271; 63.9%), the perceptions of accessibility were nearly equally divided, with 42.9% finding this delivery type accessible and 37.3% finding it inaccessible (Fig. 6). By contrast, the fewest participants rated online psychotherapy with no therapist involved as helpful (*n* = 100; 23.6%). However, 36.8% (*n* = 156) found this delivery type to be accessible. Similar to perceptions of helpfulness, most respondents were unsure about the accessibility of the different care deliveries, particularly those in online or group therapy formats. Most of the individuals who were unsure about the helpfulness or accessibility of mental health care services were not receiving medication or psychotherapy (Additional file 2).

When focusing on post-secondary mental health services, 46.9% (*n* = 196) of respondents believed they had a good understanding of the mental health resources offered by their institution. The exact number of participants (*n* = 167; 40%) were either neutral or agreed that their institution's mental health programs and services were adequate. At the same time, 73.2% (*n* = 306) of students believed that post-secondary mental health resources need to be increased. Most students (*n* = 186; 44.5%) did not feel comfortable reaching out to faculty members or staff to access support for their mental health, although most were either neutral (*n* = 137; 30.4%) or agreed (*n* = 181; 43.3%) that institutional staff promoted mental health resources. Most individuals were also neutral (*n* = 160; 38.3%) when asked if they prefer using services outside their institutions. At the same time, most participants (*n* = 187; 45.6%) did not believe they could afford private mental health care, despite 61.2% (*n* = 251) being aware of resources outside their institutions. Most participants were neutral (*n* = 172; 42%) or agreed (*n* = 157; 38.3%) that mental health services outside their campuses were more accessible. Most individuals (*n* = 186; 45.4%) also preferred using services outside of their institution, even though most were neutral (*n* = 210; 51.2%) about whether the quality of the service was superior to those on campus.

**Fig. 7:**
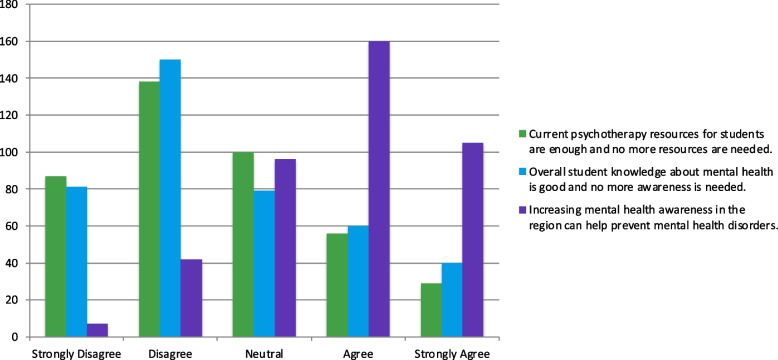
Opinions on mental health awareness and psychotherapy resources

Many students (*n* = 231; 56.5%) believed that greater awareness about mental health is necessary and that increasing awareness in the region could prevent mental health disorders (*n* = 265; 64.6%; Fig. 7). Students also believed that current psychotherapy resources were insufficient and that more resources were needed (*n* = 225; 54.9%).

**Fig. 8:**
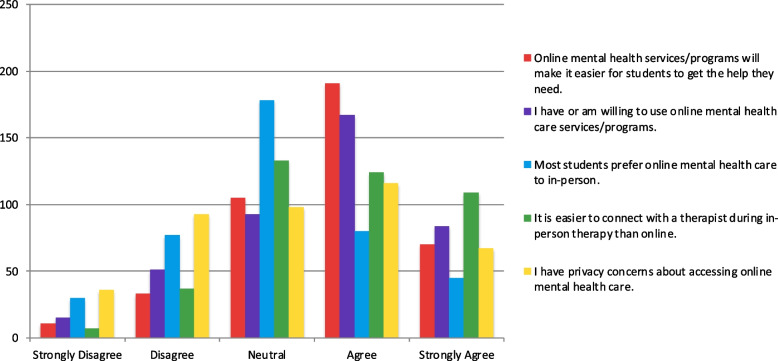
Opinions on online mental health care

Finally, when exploring the perception of online mental health care, students were relatively divided about their preference for online and in-person care, with most being neutral (*n* = 178; 43.4%; Fig. 8). At the same time, most had or were willing to use online mental health care services (*n* = 251; 61.2%). Although online mental health care was believed to make it easier for students to get the help they need (*n* = 261; 63.7%), many thought it was easier to connect with therapists in-person than online (*n* = 233; 56.8%). A large number of participants also expressed that they had privacy concerns accessing online mental health care (*n* = 183; 44.6%).

### Qualitative data

Initially, 155 participants (34.6%) responded when asked in the survey whether they had any additional feedback to share. After removing irrelevant content (i.e., responses with “no” or “not applicable” without follow-up statements), a final set of *n* = 110 (24.6%) feedback responses were coded. Agreement between the coders was moderate, κ = 0.53, (95% CI, 0.64 to 0.42), *p* < 0.001. After resolving discrepancies, seven categories and 24 subcategories emerged from the data (Table 3). Personal strategies (*n* = 35; 31.82%) and the need for mental health education (*n* = 29; 26.36%) were the most frequently expressed categories. Personal strategies were suggested by respondents and mostly stressed interpersonal skills (*n* = 9) to improve social connections, methods to enhance self-awareness (*n* = 7) and self-regulation (*n* = 5), and lifestyle changes (*n* = 5). Concerning mental health education, most feedback centred on the need to develop a formal course (*n* = 16) with content focused on personal strategies (*n* = 7). The most frequently discussed service and support focused on the need for guidance and counselling (*n* = 6). Three respondents also highlighted the institution’s role, particularly the need for support from faculty and staff. The feedback about awareness mostly centred around highlighting its importance (*n* = 10). The few environmental risk factors described digital online technology (*n* = 1), systemic factors (*n* = 2), and COVID-19 (*n* = 2). Further, changes to the post-secondary learning environment were related to improving time management by extending the years needed to complete a degree and spreading out the course workload (*n* = 2). One person also suggested improving institutional hardware and software. Lastly, participants who provided survey feedback (*n* = 11; 10%) thanked the researchers for the survey (*n* = 6) and offered suggestions to improve the survey (*n* = 4). Changes pertained to a question using the term “prevention” rather than “treatment,” including an “I don’t know” option in some parts of the survey, a technical error where a blank question was visible, and the consideration of student diversity in mental health status. A comparison of demographic data between all survey respondents and those who provided qualitative feedback demonstrated significant differences (Additional file 3). Participants who provided feedback were mostly men, male, and single (Additional file 3). Furthermore, the ratio of white to black respondents was reduced from 3.75 times amongst all survey respondents to 1.74 times in participants who provided qualitative feedback.

**Table 3:** Content analysis of open-ended feedback provided by post-secondary students (*n* = 110) at the end of the *Post-Secondary Student Mental Health Experience Survey*

Category	Sample Quote	*n* (%)
**Personal strategies**		**35 (31.82)**
Interpersonal skills	By participating in group activities, we can make many intimate friends and improve our interpersonal skills	9
Self-awareness	To maintain good mental health, we should accumulate life experience, and constantly carry out self-understanding, self-knowledge, and self-realization	7
Lifestyle changes	College students must cultivate more interests and hobbies…hobbies and interests can help adjust psychology	5
Self-regulation	Today, too many students do not know how to control themselves, resulting in serious consequences. We must learn to control ourselves and be able to regulate our emotions	5
Problem-solving	Study skills and time management skills may also help the mental health of post-secondary students	3
Avoidance behaviours	Avoid college detours and mental health problems	2
Crisis management	We should strive to improve the psychological defense mechanism, master the methods to avoid psychological crisis, and actively face life	2
Positive mindset	Keep a positive attitude at all times	2
**Mental health education**		**29 (26.36)**
Formal course	Mental health courses should be treated as formal courses, which should be valued by students and teachers	16
Educational content	Teach students to be open-minded and kind to themselves and others	7
Expert team	Improve the professional level of mental health teachers	3
Importance of education	Mental health education is the most important and direct way for college students to improve their psychological quality and promote their physical and mental health	2
Inclusivity	Mental health is not just a subject for every college student. Many school staff, including students' parents, may also need counseling on mental health. Therefore, diversified courses can be taught in the classroom to involve more people and gradually arouse interest in mental health	1
**Services and support**		**14 (12.73)**
Counselling and guidance	Students need regular access to counselling covered by their university ($200 for a 50-min session once a week is not something any student can afford)	6
Institutional role	I wish mental health and well-being is given more emphasis by faculty members for their fellow students…Mental health should be a top priority among any institution, be it educational or industry!	3
Resource information	My university needs to find a new way to inform students about these resources… They send out emails occasionally, but they aren't too great	3
Inadequacy of services	Universities provide inadequate support for graduate student mental health specifically… I would much prefer to receive therapy from an external source not associated with the university, but I am unable to do so due to lack of insurance coverage. Graduate students are often forgotten in discussions of mental health	2
**Awareness**		**13 (11.82)**
Importance of awareness	The establishment of mental health awareness is a crucial first step. Because mental health knowledge is an essential weapon for college students to improve their self-understanding and achieve self-regulation	10
Proactive measures	I think mental health is so important amongst all students. Yes, it can be a challenging topic to discuss but it would be beneficial if we talked about how to cope with mental health even as early as elementary school	2
Peer support	Mental health should involve getting to meet with students with the same issues and learning from each other	1
**Survey feedback**		**11 (10)**
Thanking for survey	Mental health awareness is much needed. Thank you for doing surveys and research in this field	6
Suggested changes	I noticed one of the boxes was "prevent mental health disorders.” Mental health disorders can never be prevented, only treated. They can also never be cured in any way; therapy is used to treat it. So could be worded better	5
**Environmental risk factors**		**5 (4.55)**
Digital and online technology	Mental illness as a whole I feel is a result of the digital age. We are never content, someone always looks better, is doing better, has more money, etc. It leads to feeling extremely unfulfilled by comparison	1
Systemic	Mental wellness in post-secondary institutions is a systemic issue. Undergraduate course loads are heavy and fast-paced, graduate/post-doc/prof research is entirely output-driven. Implementation of mental health resources is regarded as separate from academic culture rather than integrated/able into lifestyle balance	2
COVID-19	I just want to emphasise that the COVID-19 pandemic was unsurprisingly a huge factor in the decline of my mental health throughout post-secondary [school]!	2
**Curriculum restructuring**		**3 (2.73)**
Time management	Spread out the course workload…Spread out the assessments and make sure that they actually gauge the course understanding…Don't assume students know something or can learn a topic as fast as a professor	2
Hardware and software	Strengthen the guarantee of all kinds of hardware and software construction	1

## Discussion

The current study explored the mental health challenges and care needs of post-secondary students in the post-lockdown COVID-19 pandemic period (May to June 5, 2022). To capture this data, the *Post-Secondary Student Mental Health Experience Survey* was developed and made available to students within Ontario, Canada. Approximately 0.05% of the postsecondary population in the province (448 out of 903, 780 individuals [31]) responded to the survey. A decline in mental health was reported by post-secondary students since starting their studies. Although there was a greater preference for in-person treatments, many students used online mental health interventions. However, there was uncertainty about the helpfulness and accessibility of different in-person and online interventions. Several limitations to care were also expressed, including the need to improve mental health awareness, education, and care resources.

In line with the current body of studies, the most prevalent mental health disorders among students were depression and anxiety [32]. In students not formally diagnosed, the majority experienced mental health concerns and indicated a decline in their mental health since starting their post-secondary education. Indeed, post-secondary students are considered an at-risk population for chronic stress and poor mental health [33]. The current study provided additional details on how these symptoms present themselves—namely in the form of compromised concentration, symptoms of anxiety and depression, mood swings, panic attacks, problematic use of alcohol and other recreational drugs, and problems with sleep. Given the number of hours students work and engage in extracurricular activities, it is unsurprising that the majority surveyed lacked time to focus on their mental health. The pressures to meet the increased cost of living, combined with the competitiveness of the current job market, can put students at higher odds of experiencing anxiety and depression [34].

Much of the mental health needs of the sample focused on education, strategies, and tools. Although most students believed they had good mental health knowledge and offered personal strategies in the survey feedback, they could not effectively manage their concerns. These findings provide some insight into the importance of distinguishing between knowledge acquisition and knowledge use. Health knowledge can influence health behaviours and attitudes, even during the pandemic [35]. However, the current body of studies has presented caveats to the positive association between health knowledge and health behaviours. For example, one pilot study demonstrated that consumers of self-help books might present more significant symptoms of stress compared to nonconsumers [36]. In social media-based research, a gap was identified between health knowledge acquisition, intention, and health behaviours. This gap was influenced by several factors, including fear, trust, credibility, and perceived efficacy [37]. Moreover, nearly one in four students in the current study reported being subjected to some form of harassment on campus despite relevant policies, educational resources, and initiatives available to the community [38]. While it is beyond the scope of this study, future studies should examine the disconnect between knowledge and action—frequently termed the *G.I Joe Phenomena* [39]—in the context of post-secondary mental health education development. This is especially important considering that one of the most predominant feedback from participants was the need for mental health-based courses that focused on teaching personal coping strategies. In line with the theme of knowledge acquisition, most students also believed in the importance of high school mental health education. High school psychoeducation can help students transition to post-secondary school [40] by providing resources and support, enhancing student mental health literacy and hygiene, improving psychological resilience, and mitigating distorted thought processing [41, 42]. At the same time, a more robust evidence base is needed to support effective mental health promotion strategies from a young age [43]. Taken together, how and when information should be disseminated to students to evoke actionable change requires further investigation.

Regarding treatment-seeking and care needs, many students were unsure about the accessibility and helpfulness of the treatments offered. Privacy and confidentiality, communication concerns, and the quality of resources may impact acceptability and engagement with different delivery methods [44]. In addition, most of the students surveyed were not diagnosed with a mental health disorder, which may partially explain these trends. The high level of stigma that exists in this population may also limit knowledge about the resources offered. These factors further support programs that inform students of available resources. Considering the risk of developing or worsening mental health symptoms in this population, institutions may benefit from proactive measures that increase mental health awareness and knowledge of resources. Programs with these aims have successfully reduced stigma and increased resiliency and help-seeking [45]. How these approaches impact campus accommodation use and treatment-seeking are important considerations for future research [46]. Similar to other populations [47], delivery methods with therapists were more frequently perceived as accessible and helpful. The emergent categories of interpersonal skills, peer support, and counselling and guidance within the qualitative findings also support the association between human connection and mental health. Future studies should investigate whether the same benefits exist with paraprofessionals (e.g., peer supporters, lay counsellors, and other non-clinicians) since improved outcomes have been observed in digital mental health interventions that employ such personnel [48]. Despite the greater preference for in-person mental health care and increased therapist involvement, students expressed challenges in seeking help due to financial constraints, long wait times, and lack of time to focus on mental health. Online resources benefit from integrating cost-effectiveness, time flexibility, and rapid availability to alleviate some barriers to care [49, 50].

The study is one of few that investigates the mental health challenges and care needs of post-secondary students in the post-lockdown period of the COVID-19 pandemic. While the novelty of this study serves as its strength, some of its limitations must also be mentioned. A smaller region was used to pilot the survey before administering it to a larger population. Since all survey advertisements occurred through social media, there is the possibility of volunteer bias. A lack of paid advertisement may have also limited reach to include only students who access the groups and platforms the survey was advertised on. The substantial increase in technology use to connect with others can make social media a feasible tool for survey recruitment during the COVID-19 pandemic [51]. However, as students return to campus, future recruitment strategies should consider partnerships with teaching faculty and institutional and student organizations that can build trust and enhance reach amongst postsecondary students. Further, although the sample size and handling of missing data were adequate for the study’s descriptive analysis, some factors were not weighted similarly due to different response rates. While this may be a limitation, the pilot survey provided opportune conditions to re-assess the survey items and add “I don’t know,” “unsure,” or “prefer not to answer” options to discourage skipping items. A suggestion to include “does not apply” was also expressed by a participant in the qualitative feedback. The response rates were sufficient for all items, except when students were asked if they were taking medication for their mental health. When given only “yes” or “no” options, the response rate for this item was approximately 58%. Although the aforementioned options will be added to the next iteration of the survey, future studies should investigate the potential role of self-stigma on response rates within anonymous mental health surveys [52].

Nevertheless, the current findings indicated that mental health worsens throughout post-secondary education in the study population. Therefore, the survey will also be revised to include whether current mental health diagnoses were received before or after participants commenced their post-secondary studies. This information is critical in evaluating how symptoms and experiences manifest and differ in newly diagnosed patients versus those with longer experiences with mental health challenges. Another limitation of the study was that most respondents were not diagnosed with mental health disorders, and there were few members of equity-seeking and equity-deserving groups. Students who identify with these communities typically face additional stressors that increase their risk of mental health concerns [53, 54]. In addition, significant differences were observed in some of the demographic characteristics of feedback providers. Namely, men and males comprised most of the qualitative responders, whereas survey responders as a whole were mostly women and female. Furthermore, the ratio of white to black respondents was substantially reduced in the group that provided additional feedback. While not the main objective of this study, these patterns highlight the importance of designing inclusive surveys that provide a platform for traditionally less vocal groups to express their mental health needs [55, 56]. Future studies may need to implement procedures to build community trust and encourage survey completion by students in these groups. These data are critical in informing equitable mental health care and resources.

## Conclusions

The current study highlighted the need for more accessible mental health resources for students across the mental health spectrum. Institutions may benefit from developing formal courses with coping mechanisms and other tools, skills, and strategies to inspire action-based changes in students suffering from poor mental health. Financial constraints, lack of time, stigma, and long wait times are all factors that can reduce treatment-seeking and worsen mental health. Strategic methods to enhance mental health awareness and knowledge also have the potential to clarify the uncertainty that students have about the helpfulness and accessibility of different care delivery types. Although therapist guidance was viewed positively, future studies should explore how online therapist guidance can improve sentiment towards online care—a delivery type frequently used during the pandemic.

## Supplementary Information

**Additional file 1.**

**Additional file 2:** **Appendix 2.** Perceived helpfulness and accessibility of treatments in survey respondents currently receiving psychotherapy/counseling and medication for their mental health.

**Additional file 3:** **Table 4.** Demographic data of qualitative respondents. Statistical analysis compared data of qualitative respondents and all survey respondents. All *p* values are two-tailed and α = 0.05.

## Data Availability

All data generated or analysed during this study are included in this published article.
