# Supplementary material for: Mental health challenges, treatment experiences, and care needs of post-secondary students: a cross-sectional mixed-methods study
Source: BMC Public Health. 2023 Apr 6;23:655. doi: 10.1186/s12889-023-15452-x (PMC10076091; doi:10.1186/s12889-023-15452-x)
Supplement: Supplementary file 1 — Additional file 1. [file 12889_2023_15452_MOESM1_ESM.pdf]

# Demographic Information

## What gender do you identify as?

448 Responses

| Field | Choice Count |
| --- | --- |
| Woman | 267 |
| Man | 160 |
| Nonbinary | 15 |
| Two-Spirited | 0 |
| Prefer to self-describe | 2 |
| Prefer not to answer | 4 |

## What are your preferred pronouns?

448 Responses

| Field | Choice Count |
| --- | --- |
| She/Her | 253 |
| He/Him | 155 |
| They/Them | 12 |
| Other | 9 |
| Prefer not to answer | 19 |

## What is your biological sex?

448 Responses

| Field | Choice Count |
| --- | --- |
| Female | 281 |
| Male | 160 |
| Intersex | 1 |
| Prefer not to answer | 6 |

## What is your sexual orientation?

448 Responses

| Field | Choice Count |
| --- | --- |
| Straight/Heterosexual | 321 |
| Bisexual/Polysexual | 60 |
| Gay/Lesbian | 18 |
| Other | 15 |
| Asexual | 10 |
| Prefer not to answer | 24 |

## What is your race or ethnicity (select all that apply)?

448 Responses

| Field | Choice Count |
| --- | --- |
| White (e.g., European descent) | 236 |
| Black (e.g., African, African Canadian, Afro-Caribbean descent) | 63 |
| South Asian (e.g., Bangladeshi, Indian, Indo-Caribbean, Pakistani, Sri Lankan descent) | 56 |
| East Asian (e.g., Chinese, Japanese, Korean, Taiwanese descent) | 53 |
| Middle Eastern (e.g., Arab, Persian, Afghan, Egyptian, Kurdish, Lebanese, Turkish descent) | 20 |
| Latin American (e.g., Hispanic or Latin American descent) | 13 |
| Southeast Asian (e.g., Cambodian, Filipino, Indonesian, Thai, Vietnamese descent) | 11 |
| Multiethnic | 7 |
| Indigenous (e.g., First Nations, Inuk/Inuit, Métis descent) | 7 |
| Another ethnicity not listed | 6 |
| Do not know | 1 |
| Prefer not to answer | 18 |

## Do you have any disabilities (select all that apply)?

435 Responses

| Field | Choice Count |
| --- | --- |
| None | 351 |
| Neurodevelopmental disability (e.g., Autism, ADHD, FASD) | 36 |
| Chronic illnesses or health conditions that impact functioning. | 17 |
| Prefer not to answer | 11 |
| Learning Disability | 11 |
| Blind or visually impaired | 9 |
| Deaf or hard of hearing | 4 |
| Disability that impacts mobility | 3 |
| Other | 7 |

## Do you currently have any accommodations at your post-secondary institution (e.g. extra exam time, separate exam room, etc.)?

433 Responses

| Field | Choice Count |
| --- | --- |
| Yes | 144 |
| No | 267 |
| Prefer not to answer | 22 |

# What is the name of your post-secondary institution?

448 Responses

| Field | Choice Count |
| --- | --- |
| Queen's University | 80 |
| University of Toronto | 43 |
| Ontario Tech University | 38 |
| Carleton University | 37 |
| McMaster University | 37 |
| University of Waterloo | 35 |
| York University | 21 |
| Brock University | 20 |
| University of Guelph | 19 |
| University of Ottawa | 15 |
| St. Lawrence College | 12 |
| University of Western Ontario | 12 |
| University of Windsor | 11 |
| Trent University | 10 |
| Wilfried Laurier University | 9 |
| Durham College | 6 |
| Toronto Metropolitan University | 6 |
| Centennial College | 4 |
| Algonquin College | 3 |
| Conestoga College | 3 |
| George Brown College | 3 |
| Lakehead University | 3 |
| Ontario College of Art and Design University | 3 |
| Georgian College | 2 |
| Humber College | 2 |
| Laurentian University | 2 |
| Seneca College | 2 |
| University of Sudbury | 2 |
| Algoma University | 1 |
| Cambrian College | 1 |
| Canadian Memorial Chiropractic College | 1 |
| Confederation College | 1 |
| Fanshawe College | 1 |
| Mohawk College | 1 |
| Northern Ontario School of Medicine | 1 |
| Pharma-Medical Science College of Canada | 1 |

## What is your degree type?

448 Responses

| Field | Choice Count |
| --- | --- |
| Undergraduate | 291 |
| Graduate | 80 |
| Professional degree | 44 |
| Diploma | 24 |
| Other | 9 |

## What is your enrollment status?

448 Responses

| Field | Choice Count |
| --- | --- |
| Full-time | 396 |
| Part-time | 36 |
| On leave | 8 |
| Other | 8 |

## What is your student status?

448 Responses

| Field | Choice Count |
| --- | --- |
| Domestic (Canada) | 389 |
| International | 59 |

## Did you move to Ontario from another province for your post-secondary education?

388 Responses

| Field | Choice Count |
| --- | --- |
| Yes | 37 |
| No | 351 |

## What is your current housing arrangement?

445 Responses

| Field | Choice Count |
| --- | --- |
| Live at home with family/parents | 161 |
| Live with housemates | 127 |
| Live in campus residence | 75 |
| Live with my significant other | 38 |
| Live alone | 35 |
| Other | 9 |

# What is your relationship status?

448 Responses

| Field | Choice Count |
| --- | --- |
| Single, I have never been in a romantic relationship | 138 |
| Exclusive romantic relationship (not married) | 109 |
| Single, in a serious romantic relationship previously | 90 |
| Casually dating | 41 |
| Common-law (eg. cohabitation without legal marriage) | 17 |
| Married | 17 |
| Nonexclusive romantic relationship (not married) | 6 |
| Divorced/separated | 2 |
| Other | 5 |
| I prefer not to answer | 23 |

# Do you currently have paid employment?

445 Responses

| Field | Choice Count |
| --- | --- |
| Yes | 217 |
| No | 228 |

# Do you have children?

448 Responses

| Field | Choice Count |
| --- | --- |
| Yes | 11 |
| No | 437 |

# Extracurricular Activities

445 Responses

| Field | Choice Count |
| --- | --- |
| Student clubs/associations | 174 |
| None | 161 |
| Volunteer work | 127 |
| Organizations outside postsecondary institution | 39 |
| Other | 21 |

# Mental Health Information

## Do you have any formal mental health diagnoses (select all that apply)?

440 Responses

| Field | Choice Count |
| --- | --- |
| None | 270 |
| Depression | 85 |
| Generalized Anxiety Disorder | 80 |
| Social Anxiety Disorder | 51 |
| Panic Attacks | 40 |
| Attention Deficit Hyperactivity Disorder | 34 |
| Eating Disorder | 28 |
| Borderline Personality Disorder | 18 |
| Bipolar Disorder | 15 |
| Posttraumatic Stress Disorder | 10 |
| Alcohol Use Disorder | 5 |
| Other Substance Use Disorder | 2 |
| Psychosis | 1 |
| Other | 24 |

## Are you currently receiving therapy/counselling for your mental health?

445 Responses

| Field | Choice Count |
| --- | --- |
| Yes | 127 |
| No | 318 |

## If "yes" to currently receiving therapy/counselling, what type of therapy are you receiving (select all that apply)?

127 Responses

| Field | Choice Count |
| --- | --- |
| Video (e.g., Zoom) individual therapy | 66 |
| In-person individual therapy | 43 |
| Online program with support of a therapist | 24 |
| Phone | 16 |
| Video group therapy | 10 |
| In-person group therapy | 7 |
| Online program with no support from a therapist | 6 |
| Other | 2 |

## Are you currently taking any medication for your mental health?

258 Responses

| Field | Choice Count |
|---|---|
| Yes | 76 |
| No | 182 |

## Since starting your post-secondary education, have you experienced a decline in your mental health?

436 Responses

| Field | Choice Count |
|---|---|
| Yes | 290 |
| No | 102 |
| Unsure | 44 |

## Since starting your post-secondary education, have you experienced any of the following (select all that apply)?

436 Responses

| Field | Choice Count |
|---|---|
| Problems concentrating (e.g., easily distracted, not able to focus, not able to finish a task) | 288 |
| Symptoms of depression (e.g., low moods, low energy and low motivation) | 260 |
| Daily general anxiety (e.g., overthinking, increased heart rate, sweatiness, nausea) | 253 |
| Anxiety in social situations | 224 |
| Mood swings | 184 |
| Panic attacks (e.g., sudden episode of intense fear with severe physical reactions) | 147 |
| Problematic use of alcohol or other substances (including cannabis) | 59 |
| Not applicable/none of the above | 33 |
| Other | 13 |
| Prefer not to answer | 18 |

## Some of the barriers to receiving mental health care in post-secondary students in your region are (select all that apply):

424 Responses

| Field | Choice Count |
|---|---|
| Financial | 214 |
| Long wait lists | 202 |
| Lack of resources to address my needs | 165 |
| Unable to attend due to school commitments | 148 |
| Stigma | 133 |
| Cultural barriers | 108 |
| Past negative experiences with mental health services | 86 |

| | |
|---|---|
| Therapy of choice is not available (e.g., counseling vs. cognitive behavioural therapy) | 80 |
| Individual care is not available (e.g. majority of the programs are group-based) | 76 |
| There are no challenges | 35 |
| Other | 21 |

## Which of the following mental health services have you previously used during your post-secondary studies (select all that apply)?

433 Responses

| Field | Choice Count |
|---|---|
| None | 151 |
| Student wellness services offered by my post-secondary institution | 150 |
| Counseling and/or psychotherapy through video | 126 |
| Services offered outside of my post-secondary institution | 110 |
| In-person counseling and/or psychotherapy | 102 |
| Counseling and/or psychotherapy through phone | 71 |
| Online psychotherapy program with therapist support | 52 |
| Online psychotherapy program with no therapist support | 40 |
| Psychiatrist care | 36 |
| Peer-support groups | 27 |
| In-person group therapy | 27 |
| Video group therapy | 23 |
| Other | 5 |

## Which of the following strategies do you use to cope with stress (select all that apply)?

433 Responses

| Field | Choice Count |
|---|---|
| Distractions (e.g., hobbies such as art, cooking) | 305 |
| Connecting with friends | 258 |
| Food | 209 |
| Physical activity | 208 |
| Connecting with family | 195 |
| Mindfulness (e.g., meditation, breathing exercises) | 152 |
| Cognitive Techniques (e.g., working on thinking errors) | 121 |
| Alcohol | 71 |
| Cannabis/Marijuana | 60 |
| Sexual activity | 47 |
| Recreational drugs (other than cannabis) | 12 |
| Other | 20 |
| None | 13 |

# Perception of Mental Health Care

## Rate the helpfulness (e.g. the ability of the therapy/treatment to improve symptoms) of the following for post-secondary students:

424 Responses

| Field | Very Unhelpful | Somewhat Unhelpful | Unsure | Somewhat Helpful | Very Helpful |
|---|---|---|---|---|---|
| In-person counseling and/or psychotherapy | 12 | 24 | 117 | 137 | 134 |
| Video counseling/psychotherapy (e.g. Zoom) | 16 | 44 | 131 | 151 | 82 |
| Phone counseling/psychotherapy | 20 | 62 | 162 | 137 | 43 |
| Online psychotherapy program with weekly feedback from a therapist | 18 | 52 | 199 | 110 | 45 |
| Online psychotherapy program with no therapist involved | 46 | 75 | 203 | 74 | 26 |
| Group therapy | 20 | 48 | 213 | 102 | 41 |
| Psychiatrist care | 12 | 36 | 177 | 116 | 83 |

## Rate the accessibility (e.g. the ease of obtaining the therapy/treatment) of the following for post-secondary students:

424 Responses

| Field | Very Inaccessible | Somewhat Inaccessible | Unsure | Somewhat Accessible | Very Accessible |
|---|---|---|---|---|---|
| In-person counseling and/or psychotherapy | 41 | 117 | 84 | 138 | 44 |
| Video counseling/psychotherapy (e.g. Zoom) | 14 | 66 | 118 | 137 | 89 |
| Phone counseling/psychotherapy | 17 | 50 | 128 | 148 | 81 |
| Online psychotherapy program with weekly feedback from a therapist | 20 | 61 | 193 | 103 | 47 |
| Online psychotherapy program with no therapist involved | 20 | 51 | 197 | 97 | 59 |
| Group therapy | 23 | 60 | 195 | 114 | 32 |
| Psychiatrist care | 65 | 90 | 165 | 74 | 30 |

## In your opinion, how does digital mental health care compare to in-person?

424 Responses

| Field | Choice Count |
|---|---|
| Good but not as good | 132 |
| Unsure | 79 |
| Not good at all | 70 |
| Not applicable | 53 |
| No different | 52 |
| Better | 38 |

# Rate the following statements about online mental health care:

410 Responses

| Field | Strongly Disagree | Disagree | Neutral | Agree | Strongly Agree |
|---|---|---|---|---|---|
| Online mental health services will make it easier for students to get the help they need | 11 | 33 | 105 | 191 | 70 |
| I have/am willing to use online mental health care services | 15 | 51 | 93 | 167 | 84 |
| Most students prefer online mental health care to in-person | 30 | 77 | 178 | 80 | 45 |
| It is easier to connect with a therapist during in-person therapy than online | 7 | 37 | 133 | 124 | 109 |
| I have privacy concerns about online mental health care | 36 | 93 | 98 | 116 | 67 |

# Rate the following statements about mental health knowledge and status:

418 Responses

| Field | Strongly Disagree | Disagree | Neutral | Agree | Strongly Agree |
|---|---|---|---|---|---|
| Generally, my mental health knowledge is good | 11 | 47 | 77 | 202 | 81 |
| The awareness and education that I received about mental health in high school was adequate | 97 | 112 | 71 | 78 | 60 |
| Most post-secondary students have good mental health | 99 | 154 | 72 | 65 | 28 |
| I have a healthy school-personal/social life balance | 53 | 98 | 101 | 132 | 34 |
| I have a healthy level of stress in my life | 82 | 125 | 79 | 99 | 33 |
| Most students keep their mental health problems a secret | 5 | 41 | 94 | 182 | 96 |
| My mental health has worsened since starting my post-secondary studies | 17 | 47 | 110 | 127 | 117 |
| I did not have enough coping strategies and tools when I started my post-secondary studies | 18 | 61 | 78 | 164 | 97 |
| Improving high school students' mental health can enhance their functioning/wellbeing during post-secondary studies | 5 | 25 | 81 | 129 | 178 |

# Rate the following statements about mental health awareness and resources for post-secondary students:

410 Responses

| Field | Strongly Disagree | Disagree | Neutral | Agree | Strongly Agree |
|---|---|---|---|---|---|
| Current psychotherapy resources for students are enough and no more resources are needed | 87 | 138 | 100 | 56 | 29 |
| Overall student knowledge about mental health is good and no more awareness is needed | 81 | 150 | 79 | 60 | 40 |
| Increasing mental health awareness in the region can help prevent mental health disorders | 7 | 42 | 96 | 160 | 105 |

## Rate the following statements about post-secondary mental health services:

418 Responses

| Field | Strongly Disagree | Disagree | Neutral | Agree | Strongly Agree |
|---|---|---|---|---|---|
| I believe that my post-secondary institution supports student mental health | 24 | 53 | 136 | 161 | 44 |
| I have enough time to focus on my mental health during my post-secondary career | 62 | 136 | 103 | 75 | 42 |
| I have a good understanding of the mental health services offered to me by my post-secondary institution | 18 | 86 | 118 | 147 | 49 |
| I prefer using the mental health services/resources offered by my post-secondary institution than services offered outside my institution | 46 | 66 | 160 | 109 | 37 |
| My post-secondary institution does a good job promoting their mental health services | 24 | 85 | 129 | 141 | 39 |
| The mental health programs and services offered by my post-secondary institution are helpful | 19 | 65 | 167 | 128 | 39 |
| There is a need to increase mental health resources for post-secondary students | 4 | 20 | 88 | 130 | 176 |
| The mental health care available at my post-secondary institution is readily accessible | 23 | 90 | 143 | 112 | 50 |
| The waitlist to access mental health services is reasonable | 60 | 103 | 153 | 71 | 31 |
| I am afraid to use campus mental health services for fear of others finding out | 59 | 100 | 93 | 111 | 55 |
| I am comfortable reaching out to faculty members and staff (e.g. professors) to access support for my mental health | 66 | 120 | 88 | 106 | 38 |
| Faculty members (e.g. professors) at my institution promote mental health resources | 32 | 78 | 127 | 143 | 38 |
| Faculty members (e.g. professors) have a role in improving student mental health | 21 | 56 | 97 | 143 | 101 |

## Rate the following statements about mental health services off campus:

410 Responses

| Field | Strongly Disagree | Disagree | Neutral | Agree | Strongly Agree |
|---|---|---|---|---|---|
| I am aware of other mental health services/resources outside of my post-secondary institution | 26 | 68 | 65 | 183 | 68 |
| I prefer to use mental health services/resources outside of my post-secondary institution | 11 | 69 | 144 | 100 | 86 |
| I can afford private mental health care | 98 | 89 | 72 | 115 | 36 |
| The quality of mental health care offered outside of campus is better than on campus | 3 | 23 | 210 | 106 | 68 |
| The mental health care offered outside of campus is more accessible | 19 | 62 | 172 | 102 | 55 |
