# Supplementary material for: Mental health challenges, treatment experiences, and care needs of post-secondary students: a cross-sectional mixed-methods study
Source: BMC Public Health. 2023 Apr 6;23:655. doi: 10.1186/s12889-023-15452-x (PMC10076091; doi:10.1186/s12889-023-15452-x)
Supplement: Supplementary file 3 — Additional file 3: Table 4. Demographic data of qualitative respondents. Statistical analysis compared data of qualitative respondents and all survey respondents. All p values are two-tailed and α = 0.05. [file 12889_2023_15452_MOESM3_ESM.pdf]

Table 4. Demographic data of qualitative respondents. Statistical analysis compared data of qualitative respondents and all survey respondents. All p values are two-tailed and $\alpha = 0.05$

Category	n (%)	Statistical Analysis
**Gender Identity**		
Man	64 (58.2)	$\chi^2(15)=63.39$
Woman	45 (40.9)	$p < 0.001$
Prefer to self-describe	1 (0.9)	
**Biological Sex**		
Male	64 (58.2)	$\chi^2(8)=51.85$
Female	46 (41.8)	$p < 0.001$
**Sexual Orientation**		
Straight/Heterosexual	101 (91.8)	$\chi^2(30)=68.01$
Bisexual/Polysexual	6 (5.5)	$p = 0.002$
Asexual	1 (0.9)	
Other	1 (0.9)	
Prefer not to answer	1 (0.9)	
**Preferred Pronouns**		
He/Him	65 (59.1)	$\chi^2(15)=255.28$
She/Her	43 (39.0)	$p < 0.001$
Prefer not to answer	2 (1.8)	
**Race/Ethnicity**		
White (European descent)	59 (53.6)	$\chi^2(275)=62.98$
Black (African, African Canadian, Afro-Caribbean Descent)	34 (30.9)	$p = 0.798$
South Asian (Bangladeshi, Indian, Indo-Caribbean, Pakistani, Sri Lankan descent)	9 (8.2)	
East Asian (Chinese, Japanese, Korean, Taiwanese Descent)	2 (1.8)	
Middle Eastern (Arab, Persian, Afghan, Egyptian, Kurdish, Lebanese, Turkish descent)	2 (1.8)	
Southeast Asian (Cambodian, Filipino, Indonesian, Thai, Vietnamese descent)	2 (1.8)	
Indigenous (First Nation, Inuk/Inuit, Métis descent)	1 (0.9)	
Multiethnic	1 (0.9)	
**Relationship Status**		
Single, never been in a romantic relationship	39 (35.5)	$\chi^2(100)=133.11$
Single, in a serious romantic relationship previously	25 (22.7)	$p = 0.015$
Exclusive romantic relationship (not married)	19 (17.3)	
Casually dating	16 (14.5)	
Other	3 (2.7)	
Married	2 (1.8)	
Common-law	2 (1.8)	
Prefer not to answer	2 (1.8)	
Nonexclusive romantic relationship (not married)	1 (0.9)	
Divorced	1 (0.9)	
**Age (Mean, SD)**	21.65 (4.05)	$t(555) = -0.59$ $p = 0.56$
